# Levels of glycosaminoglycans in the cerebrospinal fluid of healthy young adults, surrogate-normal children, and Hunter syndrome patients with and without cognitive impairment

**DOI:** 10.1016/j.ymgmr.2015.11.001

**Published:** 2015-11-09

**Authors:** Christian J. Hendriksz, Joseph Muenzer, Adeline Vanderver, Jonathan M. Davis, Barbara K. Burton, Nancy J. Mendelsohn, Nan Wang, Luying Pan, Arian Pano, Ann J. Barbier

**Affiliations:** a The Mark Holland Metabolic Unit, Salford Royal NHS Foundation Trust, Salford M6 8HD, United Kingdom; b Department of Pediatrics, University of North Carolina at Chapel Hill, Chapel Hill, NC 27599, USA; c Center for Genetic Medicine Research, Children's Research Institute, Children's National Health System, Washington, DC, 20010, USA; d Division of Newborn Medicine, Floating Hospital for Children at Tufts Medical Center, Boston, MA 02111, USA; e Division of Genetics, Birth Defects and Metabolism, Northwestern University Feinberg School of Medicine, Chicago, IL 60611, USA; f The Ann & Robert H. Lurie Children's Hospital, Chicago, IL 60611, USA; g Department of Medical Genetics, Children's Hospitals and Clinics of Minnesota, Minneapolis, MN 55404, USA; h Division of Medical Genetics, Department of Pediatrics, University of Minnesota, Minneapolis, MN 55454, USA; i Shire, 300 Shire Way, Lexington, MA 02421, USA

**Keywords:** Mucopolysaccharidosis II, Lysosomal storage disease, Cerebrospinal fluid, Glycosaminoglycans, Idursulfase, Cognitive impairment, Inherited metabolic disease

## Abstract

In mucopolysaccharidoses (MPS), glycosaminoglycans (GAG) accumulate in tissues. In MPS II, approximately two-thirds of patients are cognitively impaired. We investigated levels of GAG in cerebrospinal fluid (CSF) in different populations from four clinical studies (including NCT00920647 and NCT01449240). Data indicate that MPS II patients with cognitive impairment have elevated levels of CSF GAG, whereas those with the attenuated phenotype typically have levels falling between those of the cognitively affected patients and healthy controls.

## 1 Introduction

Mucopolysaccharidosis II (MPS II; Hunter syndrome) is an X-linked lysosomal storage disorder (LSD) caused by a deficiency in the enzyme iduronate-2-sulfatase (EC 3.1.6.13), leading to the accumulation of glycosaminoglycans (GAGs) in lysosomes [1]. Affected patients have elevated levels of GAGs in serum, urine, and cerebrospinal fluid (CSF) [2], [3], [4].

All patients with MPS II experience a variety of somatic signs and symptoms associated with significant morbidity and early mortality [5], [6]. Approximately two-thirds of patients also display progressive cognitive decline (severe phenotype), whereas one-third of patients remain cognitively intact (attenuated phenotype) [7].

Idursulfase (Elaprase®, Shire, Lexington, MA, USA), a recombinant human iduronate-2-sulfatase, has been approved as an intravenous (IV) medicinal product for the treatment of MPS II since 2006 in the United States and 2007 in Europe [8]. Intravenous idursulfase does not cross the blood–brain barrier at the therapeutic dose and is not expected to alter the cognitive decline seen in the severe phenotype [9].

Currently, limited data are available that describe the biomarker composition of CSF [10] and quantify levels of disease-related biomarkers present in CSF of patients with lysosomal storage diseases [11], [12], [13]. Ongoing clinical trials are exploring drug delivery directly into the CSF as a way of bypassing the blood–brain barrier [14].

In four studies, we investigated the levels of GAGs in the CSF to establish ranges in four types of population: healthy adults, surrogate-normal children (children who underwent a lumbar puncture for a clinical indication other than suspicion of a lysosomal storage disease), MPS II patients with cognitive impairment, and MPS II patients with no cognitive impairment.

## 2 Material and methods

The data presented here are from four studies:

1. A phase I-II, multicenter, randomized, open-label, interventional study of MPS II patients with cognitive involvement (HGT-HIT-045, NCT00920647).The study investigated an idursulfase formulation designed for intrathecal administration (idursulfase-IT). Patients were aged < 18 years, with cognitive impairment due to MPS II (IQ < 77, or IQ decline of 15–30 IQ points in past 3 years). CSF samples were obtained at screening (prior to exposure to investigational treatment) [15].
2. A multicenter study of pediatric and adult patients with MPS II (HGT-HIT-072, NCT01449240). All pediatric patients were scheduled to undergo a non-study-related lumbar puncture or other medical or diagnostic procedure that required the administration of general anesthesia during which a lumbar puncture could be performed [16].
3. A single-center study of normal healthy adult volunteers undergoing lumbar puncture (HGT-HIT-073). Patients were ≥ 18 and ≤ 30 years of age, and with no clinically significant findings.
4. Samples from surrogate-normal children (children from whom a CSF sample was taken for a clinical indication other than suspicion of, or in the context of, a lysosomal storage disease) were obtained from two sources. One was a multicenter, noninterventional study of male or female pediatric subjects (< 18 years of age) undergoing a procedure allowing access to CSF for other clinical reasons (HGT-HIT-083). Age groups included newborns (0–27 days), infants and toddlers (28 days to 23 months), children (2–11 years), and adolescents (12 to < 18 years). The second source was 157 samples from surrogate-normal children obtained from a biorepository at The Children's National Medical Center (CNMC) in Washington DC, and these were analyzed with the HGT-HIT-083 samples.

Informed consent was obtained from all patients and/or legal guardians and independent review board/independent ethics committees approved all studies. Study procedures complied with Good Clinical Practice as described in the 21 CFR Parts 50, 56, and 312 and the International Conference on Harmonisation Guidelines, as well as with the ethical principles described in the Declaration of Helsinki.

### 2.1 CSF assessment

CSF samples were evaluated for glucose, protein, albumin, and cell counts. CSF samples were stored frozen for subsequent analysis of total GAGs. Total CSF GAG concentration was determined by Shire (Lexington, MA, USA) using a thrombin activity assay with chromogenic substrate S-2238™ (Chromogenix, Bedford, MA, USA). CSF samples were pre-incubated with a fixed amount of human heparin cofactor II (Haematologic Technologies Inc. Essex Junction, VT, USA), then incubated with a fixed amount of Human α-Thrombin (Enzyme Research laboratories, South Bend, IN, USA) and 0.5 mM S-2238 in assay buffer. GAG in CSF samples binds to heparin cofactor II, which in turn accelerates thrombin inactivation. The GAG concentration was calculated from a calibration curve prepared from dermatan sulfate (GAG-DS01™, Iduron, Manchester, United Kingdom) included in the same assay. The lower limit of quantification was 36.7 ng/mL. The assay mainly detected dermatan sulfate and heparan sulfate in the GAG family. This assay was used to measure the CSF GAG levels for all studies reported in this paper. The quantification of urinary GAG for HIT-045, HIT-072 and HIT-073 was performed by Cambridge Biomedical Inc. (Boston, MA, USA) using a Blyscan™ Glycosaminoglycan Assay kit (Biocolor Life Science Assays, Carrickfergus, United Kingdom), and results were normalized to urine creatinine [15], [16].

### 2.2 Statistical methods

Descriptive summaries were presented for the following subject groups: surrogate-normal children, healthy adults, cognitively-intact MPS II patients, and cognitively-impaired MPSII patients. Data for surrogate-normal children from study HGT-HIT-083 were merged with those from the biorepository samples and analyzed in 4 age groups.

## 3 Results

Two hundred and fifty seven subjects from the 4 studies were assessed, and their baseline or Day 1 characteristics were measured. Data from study HGT-HIT-072, which enrolled both cognitively intact and cognitively impaired children with MPS II, as well as cognitively intact adults with MPS II are not shown separately, but per cognitive category. For instance, the data from the 3 cognitively impaired children whose CSF was obtained in study HGT-HIT-072 (previously published [16]), were analyzed together with the data from the 16 cognitively impaired boys with MPS II from the phase I–II idursulfase-IT study (HGT-HIT-045)(previously published [15]), leading to a total number of 19 data points for the category of cognitively affected children with MPS II between the ages of 2–11 years.

**Fig. 1:**
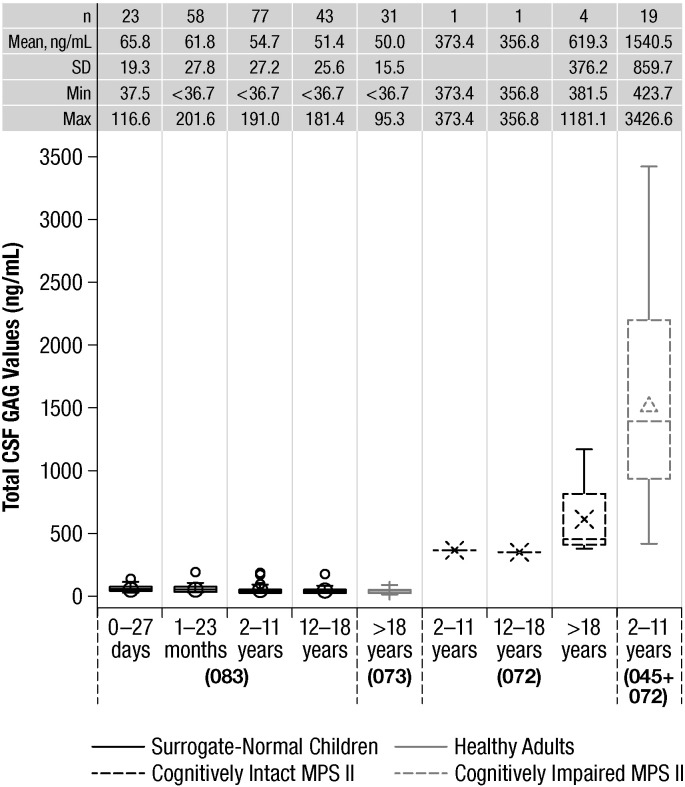
Box plots of total CSF GAG for each subject group. Notes: 1. Surrogate normal data includes data from both the 083 study and biorepository data. 2. Values < 36.7 ng/mL are below the lower limit of quantification. Values < 36.7 ng/mL are replaced with values of 36.7 ng/mL for the plot. CSF, cerebrospinal fluid; GAG, glycosaminoglycan; MPS II, mucopolysaccharidosis II; 045, HGT-HIT-045; 072, HGT-HIT-072; 073, HGT-HIT-073; 083, HGT-HIT-083.

Box plots of CSF GAG data for each subject group are shown in Fig. 1. The average GAG level in healthy adults (n = 31) was 50.0 ± 15.5 ng/mL (mean ± SD). In surrogate-normal children, the mean GAG levels for neonates were 65.8 ± 19.3 ng/mL (n = 23), for 1–23 months 61.8 ± 27.8 ng/mL (n = 58), for 2–11 years 54.7 ± 27.2 ng/mL (n = 77), and for 12–18 years old 51.4 ± 25.6 ng/mL (n = 43). These data indicate that GAG levels in non-MPS II subjects were all below approximately 200 ng/mL, with means in the range of 50–70 ng/mL depending on age. We obtained samples from two pediatric and four adult patients with MPS II without cognitive impairment, and found that the two pediatric patients had values of 356.8 and 373.4 ng/mL. Of the four adult patients with the attenuated MPS II phenotype, three had levels in the 380–460 ng/mL range and one had a value of 1181.1 ng/mL. Samples from the 19 cognitively impaired children aged 2–11 years (representing the severe phenotype of MPS II) had average CSF GAG levels of 1540.5 ± 859.7 ng/mL. The lowest value observed in a cognitively impaired MPS II boy was 423.7 ng/mL.

Urinary GAG Levels were measured in studies HGT-HIT-045, HGT-HIT-072, and HGT-HIT-073. In the HGT-HIT-072 study, all 9 patients were receiving intravenous idursulfase treatment, therefore urinary GAG levels did not reflect true baseline levels in these patients. In the HGT-HIT-073 study the adult normal subjects had urinary GAG concentrations that were within 2 times the upper limit of normal (ULN, < 5.50 mg GAG/mmol creatinine). In the HGT-HIT-045 study all patients had received and tolerated a minimum of 6 months of treatment with intravenous idursulfase, prior to starting the study, so urinary GAG levels would not be expected to reflect accurately any baseline levels. In the HGT-HIT-083 study, no other GAG measurements were taken, as the study activities were limited to obtaining samples of CSF. None of studies measured serum GAG levels. For these reasons there is insufficient data to perform correlation analyses of urinary GAG and CSF GAG levels in these studies.

## 4 Conclusions

Dekaban and his colleagues initially reported in 1973 that subjects with MPS and unimpaired intellects had lower levels of CSF GAGs than MPS subjects who were mentally impaired [17]. Our study expands on this and on the Hendriksz et al. study that reported differences between pediatric and adult MPS II patients' CSF GAG levels (the data from that paper is included here) [16]. This is the first study to undertake a systematic exploration of CSF GAG levels in MPS II patients and to compare them to healthy individuals. Our data show that healthy adults or surrogate-normal children (children who have had a lumbar puncture for clinical indications other than suspicion of MPS) have CSF GAG levels that are, on average, 50–70 ng/mL, depending on age, and almost always below 200 ng/mL. In contrast, we found that MPS II patients with cognitive impairment had markedly elevated levels of CSF GAG, with levels between approximately 400 and 3500 ng/mL and averaging close to 1500 ng/mL. The values observed in both pediatric and adult patients with the attenuated MPS II phenotype always exceeded those observed in non-MPS individuals, but overlapped with those observed in the cognitively affected MPS II patients. These data suggest that CSF GAG levels may be useful diagnostic and prognostic tools for the classification and management of patients with MPS II, including the earlier identification of patients at risk for developing cognitive impairment. It is too early, however, to be able to make concrete suggestions as to what levels of GAG in the CSF correlate with what degree of cognitive impairment, and further data is needed to confirm and extend the analysis.

## Financial disclosures

Dr. Christian Hendriksz has received consulting fees from Actelion, BioMarin, Glaxo-SmithKline, and Sanofi-Genzyme, and has undertaken contracted research for Actelion, Amicus, BioMarin, GlaxoSmithKline, Sanofi-Genzyme, Shire, and Synageva. Dr. Joseph Muenzer has been a consultant to BioMarin, Shire, and Zacharon, and serves on advisory boards and speakers bureaus for Genzyme, BioMarin, and Shire. He is also currently the principal investigator for phase 1/2 and phase 2/3 IT ERT clinical trials for MPS II sponsored by Shire. Dr. Adeline Vanderver has received consulting fees from Shire and has performed (unpaid) consulting for Stemcells, Inc. Dr. Jonathan Davis has no conflicts to declare. Dr. Barbara Burton has received consulting fees from, and serves on advisory boards for, BioMarin and Shire, and has undertaken contracted research for BioMarin, Cytonet, Genzyme, Shire, Synageva, and Ultragenyx. Dr. Nancy Mendelsohn has received grant funding from Genzyme, BioMarin, and Shire. Ms. Nan Wang and Dr. Luying Pan are employees of, and own stock in Shire. At the time of these studies Drs. Arian Pano and Ann Barbier were employees of Shire and they own stock in Shire.
